# Trends in Colorectal Cancer Incidence Rates in Saudi Arabia (2001–2016) Using Saudi National Registry: Early- Versus Late-Onset Disease

**DOI:** 10.3389/fonc.2021.730689

**Published:** 2021-09-09

**Authors:** Mesnad Alyabsi, Mohammed Algarni, Kanan Alshammari

**Affiliations:** ^1^Population Health Research Section, King Abdullah International Medical Research Center, Riyadh, Saudi Arabia; ^2^King Saud bin Abdulaziz University for Health Sciences, Riyadh, Saudi Arabia; ^3^Oncology Department, Ministry of National Guard—Health Affairs, Riyadh, Saudi Arabia; ^4^King Abdullah International Medical Research Center, Riyadh, Saudi Arabia

**Keywords:** colorectal cancer, Saudi Arabia, registry, incidence rate, population-based

## Abstract

Early-onset (<50 years old) colorectal cancer (CRC) has been increasing worldwide and is associated with poor outcomes. Over 85% of the Saudi population are <50 years old, which put them at heightened risk of early-onset CRC. No study assessed the trends in CRC incidence rates among the Saudis. The Joinpoint Regression software by the Surveillance, Epidemiology, and End Results (SEER) program was used to estimate the magnitude and direction of CRC incidence trends by age and gender. The annual percentage change (APC) and the average annual percentage change (AAPC) between 2001 and 2016 were computed. In a sensitivity analysis, we also assessed trends using various age groups. Between 2001 and 2016, the early-onset CRC incidence (per 10^5^) increased from 1.32 (95% CI: 1.11, 1.54) to 2.02 (95% CI: 1.83, 2.22) with AAPC (2.6, 95% CI: -0.4, 5.7). At same period, the late-onset incidence increased from 3.54 (95% CI: 3.10, 3.97) to 9.14 (95% CI: 8.62, 9.66) with AAPC (6.1, 95% CI: 3.5, 8.8). Among early-onset CRC patients, age 40–49 has the highest rates and women in this age group has higher rate than men. Our national data showed a gradual increase in CRC incidence rates, which reflect the global concern of early-onset CRC. Further research is needed to understand the etiology of early-onset CRC. Primary health care providers must be alerted about the increasing rate of early-onset CRC. To reduce the future burden of the disease, initiating CRC screening before age 50 is warranted.

## Highlights

Given the global concern of increasing incidence rates of early-onset colorectal cancer, this cross-sectional analysis of data between 2001 and 2016 showed an increase the CRC incidence rate in Saudis including the early-onset colorectal cancer. This finding raise questions about the importance of initiating screening in individuals younger than 50 years old.

## Introduction

Colorectal cancer (CRC) is the third most diagnosed cancer globally with more than 1.9 million incident cases in 2020 (1). While the global incidence rates have been decreasing in the screening-eligible age group (50–75) due to the adoption of CRC screening and reduction in risk factors such as smoking, there have been global reports of increasing rates in the younger population (<50), with the highest annual percentage change (APC) among the age group 20–39 (2, 3). These reports bring about the discussion about the appropriate age to initiate the screening, with some reports advocating for starting at age 45 and others as early as the age of 40, after considering the benefit-risk profile of screening at younger age (4–6).

Unlike other international studies, investigating the trends of CRC among the Saudi population is critical for several reasons. First, the Saudi population is young with 35% in the age group 20–39 and 86% younger than 50 years old (7). Second, obesity is common among the Gulf Cooperation Council (GCC) countries, and Saudi Arabia is no exception. In 2016, 35% of Saudi adults and one in five adolescents are considered obese (8). Both, a young population with a high proportion of obesity are characteristics that engender the development of early-onset CRC. Studies from the US, Canada, the UK, Australia, and New Zealand showed an association between the childhood obesity epidemic and the rise in early-onset CRC (9). For instance, women with BMI ≥ 23 at age 18 had a 63% higher risk of early-onset CRC compared to women with a BMI of 18.5–20.9 (10). Third, there is currently no population-based screening for CRC in Saudi Arabia, leading to delayed CRC detection, increased late-stage diagnosis, and poor survival across all age groups (11–13).

In Saudi Arabia, CRC is the most diagnosed cancer in men and third in women with 1659 cases reported in 2016, representing almost 13% of all diagnosed cancers (14). The age-standardized incidence rates per 10^5^ people during 2016 were 12.9 and 9.5 in males and females. It is, nevertheless, unknown if the incidence rates have been increasing similarly across all age groups, and no study has investigated changes in incidence rates by age at diagnosis. While approximately 13% of early-onset CRC develops from germline mutations in genes causing hereditary CRC syndromes, the majority of early-onset CRC are sporadic, poorly differentiated, with mucinous adenocarcinoma and are diagnosed at late stage (15, 16).

On May 18, 2021 and in an effort to detect the disease at early stages, the United State Preventive Services Task Force (USPSTF) recommended CRC screening in adults aged 45–49 years with a grade “B” recommendation (17). While the recommendation recognizes the aggressive nature of early-onset CRC (4, 6), it also reflects the implications of early-onset CRC in terms of the choice of therapies and prognosis. Accordingly, it is imperative to characterize age groups with the heightened risk of early-onset CRC in the Saudi population and to investigate CRC trends in this young population. Therefore, the specific aims of the present study were to assess the average annual incidence rates for the years 2012–2016, to assess the time-weighted average annual percentage change (AAPC) during the recent 10 years (2007–2016) and 5 years (2012–2016), and to compare the incidence rates by age, gender, and subsites.

## Materials and Methods

### Study Design and Data Source

The Saudi Cancer Registry (SCR) was used in this study. The SCR is a population-based cancer registry that was established in 1992 and collects all cancer cases in Saudi Arabia. The registry gathers information using CanReg that meets high-quality cancer registration set by the International Agency for Research on Cancer (IARC). For the current study, we retrieved all CRC cases diagnosed between 2001 and 2016. Data were retrieved from patients’ medical records using clinical and histopathological diagnoses through trained cancer registrars. To ensure completeness and validity of the data, the tumor’s information is reviewed, coded using the International Classification of Diseases for Oncology 3^rd^ Edition (ICD-O-3), and then linked from various regions. Census data of the Saudi population were obtained from the General Authority for Statistics.

### Covariates and Outcome Variables

The primary outcome was the average annual incidence rates during 2001–2016, 2007–2016, and 2012–2016. The incidence rates were stratified by age at diagnosis and categorized as <50 (early-onset CRC) or ≥50 years (other younger age categories were also reported). The subsite for CRC was also categorized according to the ICD-10 codes with colon cancer (code C18) and rectal cancer (code C19 and C20). The 13 regional areas of Saudi Arabia have also been retrieved from the Saudi Authority of Statistics as well as the SCR and were used in the calculation of rates.

### Sensitivity Analysis

According to Jacobs et al. (18), there is a distinction between the early-onset colon (20–44 years) and rectal cancers (≤54 years). Based on this difference, the authors suggest different definitions for eligibility criteria among early-onset colon and rectal cancer patients. Therefore, we categorize the CRC patients based on the authors’ suggestions. Additionally, to compare our results with global research (3, 19), we have also investigated the trends in incidence rate among the age group 20–49.

### Statistical Analysis

Incidence rates were computed annually and were averaged over the entire study period to examine changes over time. The rates were computed by dividing the age-specific number of incident CRC cases by the appropriate age-specific person-years at risk, as determined from the General Authority for Statistics stratified by gender. The exact Poisson 95% confidence intervals for these rates were calculated in SAS version 9.4 (SAS Institute, Cary, NC). The rates are reported per 10^5^ population and were age-standardized using the world standard population.

The time-weighted AAPC was also computed using the Surveillance, Epidemiology, and End Results Program (SEER) Joinpoint regression analysis. The method fits joined straight lines to the observed age-adjusted incidence rates on a logarithmic scale (20). The method tests the null hypothesis of a zero joinpoint against the alternative hypothesis of maximum joinpoints. The maximum joinpoints are determined by the total number of years available in the registry. If the AAPC is statistically significantly different from zero (p < 0.05), then trends are considered increasing or decreasing; otherwise, they are considered stable trends.

## Results

**Table 1** shows the characteristics of the Saudi population and CRC cases from the most recent available data during the year 2016. The majority (39%) of the population is younger than 20 years old, about 86% is younger than 50 years old, and mostly reside in regions of Riyadh, Makkah, and Eastern province. Almost one-third of the CRC cases are among age groups 40–54, are predominantly males, and reside in the three most populated regions. While there has been an increase in the age-standardized rates across all age groups, the steepest increase was among patients age 50 years or older (**Table 2**).

**Table 1:** Characteristics of the Saudi population and the population-based colorectal cancer cases, Saudi Arabia, 2016.

Characteristics	Saudi population, 2016 (n = 20,064,970)		CRC cases, 2016 (n = 1654)	
	N	%	N	%
**Age**				
<20	7849953	39.12	2	0.12
20–29	3888427	19.38	28	1.69
30–39	3219098	16.04	120	7.26
40–49	2314483	11.53	270	16.32
50–54	838595	4.18	237	14.33
55–59	644701	3.21	212	12.82
60–64	471268	2.35	218	13.18
65–69	315851	1.57	148	8.95
70–74	211897	1.06	154	9.31
75+	310697	1.55	265	16.02
**Gender**				
Male	10225650	50.96	950	57.44
Female	9839320	49.04	704	42.56
**Region**				
Asir	1719950	8.57	148	8.96
Baha	376204	1.87	21	1.27
Jazan	1187284	5.92	32	1.94
Madinah	1353102	6.74	92	5.57
Hail	529012	2.64	35	2.12
Qassim	991032	4.94	77	4.66
Najran	430711	2.15	11	0.67
Jouf	373662	1.86	21	1.27
Tabuk	710699	3.54	28	1.70
Northern region	285486	1.42	14	0.85
Riyadh	4579570	22.82	501	30.35
Makkah	4440571	22.13	359	21.74
Eastern province	3087687	15.39	312	18.90

**Table 2:** Colorectal cancer age-adjusted incidence rates by age group and year, Saudi Arabia, 2001–2016.

Year	<50 years		50+ years		All ages	
	ASR	95% CI	ASR	95% CI	ASR	95% CI
2001	1.32	(1.11, 1.54)	3.54	(3.10, 3.97)	4.86	(4.38, 5.35)
2002	1.44	(1.22, 1.66)	3.98	(3.53, 4.42)	5.41	(4.92, 5.91)
2003	1.35	(1.14, 1.56)	5.22	(4.70, 5.74)	6.57	(6.01, 7.13)
2004	1.56	(1.33, 1.78)	5.77	(5.23, 6.31)	7.33	(6.74, 7.92)
2005	1.79	(1.56, 2.03)	6.66	(6.09, 7.23)	8.45	(7.84, 9.07)
2006	1.58	(1.37, 1.79)	7.58	(6.93, 8.22)	9.15	(8.47, 9.83)
2007	1.96	(1.73, 2.19)	6.89	(6.34, 7.45)	8.86	(8.26, 9.46)
2008	1.80	(1.58, 2.02)	6.82	(6.28, 7.36)	8.62	(8.03, 9.20)
2009	2.13	(1.89, 2.37)	8.40	(7.80, 9.00)	10.53	(9.89, 11.17)
2010	1.99	(1.76, 2.22)	7.55	(6.99, 8.11)	9.54	(8.94, 10.14)
2011	2.01	(1.79, 2.22)	8.08	(7.53, 8.63)	10.09	(9.49, 10.68)
2012	1.85	(1.65, 2.05)	8.23	(6.69, 8.78)	10.08	(9.50, 10.67)
2013	1.74	(1.56, 1.92)	7.67	(7.19, 8.14)	9.41	(8.89, 9.92)
2014	1.64	(1.46, 1.82)	7.56	(7.08, 8.04)	9.20	(8.69, 9.71)
2015	1.64	(1.46, 1.82)	8.29	(7.79, 8.78)	9.93	(9.40, 10.45)
2016	2.02	(1.83, 2.22)	9.14	(8.62, 9.66)	11.17	(10.61, 11.72)

**Table 3** displays the average age-standardized incidence rates during the years 2007–2016 and the years 2012–2016. Overall, the incidence rates are similar in the most recent 5 years (2012–2016) and the past 10 years (2007–2016). In general, men have higher rates than women except in the age groups 40–49, where women have higher rates than men. Among patients with early-onset CRC (<50), those in the age group 40–49 have the highest rates and even higher than those 50–54 and 55–59, especially among women.

**Table 3:** Age-standardized colorectal cancer incidence rates during the most recent years by gender, Saudi Arabia.

Age	Men		Women		Both	
	2007–2016	2012–2016	2007–2016	2012–2016	2007–2016	2012–2016
	Incidence rate (95% CI)	Incidence rate (95% CI)	Incidence rate (95% CI)	Incidence rate (95% CI)	Incidence rate (95% CI)	Incidence rate (95% CI)
<20	0.02 (0.02, 0.03)	0.02 (0.01, 0.03)	0.02 (0.01, 0.03)	0.02 (0.01, 0.04)	0.04 (0.03, 0.05)	0.04 (0.03, 0.05)
20–29	0.14 (0.13, 0.16)	0.13 (0.10, 0.16)	0.13 (0.11, 0.14)	0.12 (0.09, 0.15)	0.27 (0.25, 0.28)	0.26 (0.23, 0.27)
30–39	0.39 (0.37, 0.41)	0.39 (0.34, 0.43)	0.41 (0.38, 0.42)	0.41 (0.35, 0.45)	0.79 (0.76, 0.82)	0.80 (0.75, 0.82)
40–49	1.23 (1.18, 1.28)	1.25 (1.11, 1.31)	1.40 (1.30, 1.42)	1.31 (1.17, 1.38)	2.58 (2.52, 2.63)	2.56 (2.41, 2.58)
50–54	1.13 (1.09, 1.18)	1.17 (1.07, 1.28)	1.15 (1.10, 1.20)	1.16 (1.06, 1.27)	2.28 (2.23, 2.34)	2.34 (2.26, 2.41)
55–59	1.36 (1.31, 1.42)	1.51 (1.38, 1.63)	1.12 (1.06, 1.18)	1.15 (1.04, 1.26)	2.49 (2.42, 2.55)	2.65 (2.57, 2.74)
60–64	1.65 (1.58, 1.73)	1.77 (1.61, 1.93)	1.44 (1.36, 1.51)	1.53 (1.38, 1.67)	3.09 (3.01, 3.17)	3.30 (3.19, 3.40)
65–69	1.82 (1.74, 1.89)	1.69 (1.52, 1.85)	1.26 (1.18, 1.33)	1.33 (1.19, 1.47)	3.08 (2.99, 3.16)	3.02 (2.91, 3.13)
70–74	1.48 (1.42, 1.55)	1.73 (1.57, 1.89)	1.01 (0.95, 1.07)	1.04 (0.91, 1.16)	2.49 (2.42, 2.57)	2.77 (2.67, 2.87)
75+	1.66 (1.60, 1.71)	1.81 (1.67, 1.95)	1.06 (1.01, 1.12)	1.09 (0.99, 1.20)	2.72 (2.66, 2.78)	2.90 (2.81, 2.99)
0–75+	10.89 (10.44, 11.35)	11.42 (10.38, 12.46)	8.94 (8.48, 9.40)	9.12 (8.18, 10.06)	19.84 (19.32, 20.35)	20.54 (19.84, 21.24)
20–49	1.76 (1.68, 1.84)	1.73 (1.55, 1.91)	1.88 (1.80, 1.96)	1.80 (1.61, 1.98)	3.64 (3.54, 3.74)	3.52 (3.40, 3.65)
<50	1.78 (1.70, 1.87)	1.75 (1.56, 1.94)	1.90 (1.81, 1.99)	1.82 (1.63, 2.02)	3.68 (3.58, 3.79)	3.57 (3.43, 3.71)
50+	9.11 (8.74, 9.48)	9.67 (8.82, 10.53)	7.04 (6.67, 7.41)	7.30 (6.55, 8.04)	16.15 (15.74, 16.57)	16.97 (16.41, 17.54)

**Figure 1:**
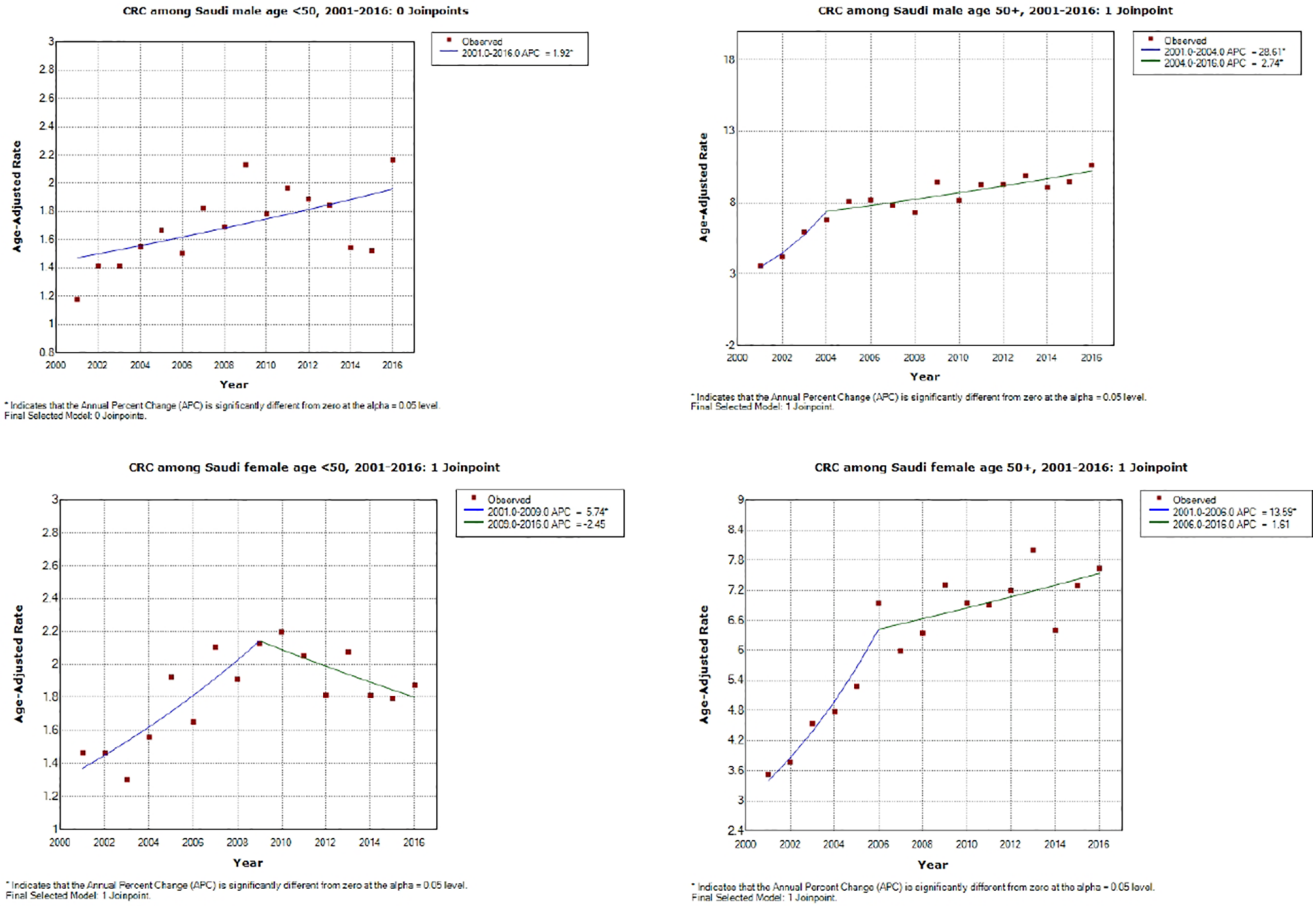
Colorectal cancer incidence trends during the study period (2001–2016) by age and gender. *APC is statistically significantly different from zero using a two-sided test based on the permutation method.

**Figure 2:**
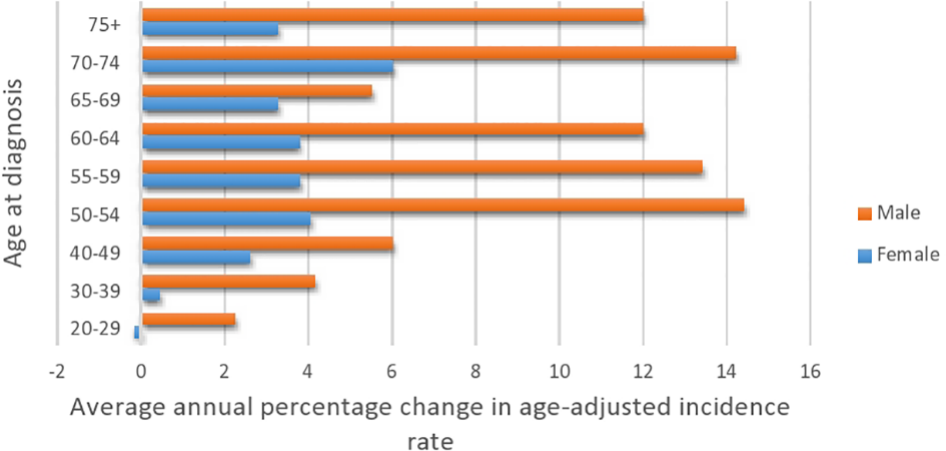
Average annual percentage change in colorectal cancer by age at diagnosis and gender (2001–2016).

As shown in **Table 4** (and **Figures 1**, **2**), both early-onset CRC patients (<50) and those 50+ have shown increased AAPC during the study period. Specifically, in the latest 5 and 10 years of data, colon cancer has shown consistent increase in 50+ patients, while it was either increasing or stable in rectal cancer patients across all age groups.

**Table 4:** Trends in colorectal cancer incidence rates by gender, age, and subsite, Saudi Arabia, 2001–2016.

Gender	Subsite	Age	Trend 1		Trend 2		Trend 3		AAPC (95% CI)	
			Years	APC (95% CI)	Years	APC (95% CI)	Years	APC (95% CI)	2001–2016	2007–2016
Male	Colon	<50	–	–	–	–	–	–	2.56 (0.8,4.3)	1.07 (-2.2,4.5)
		50+	2001–2005	22.1 (8.9, 36.9)	2005–2016	3.3 (1.8, 4.8)	–	–	8.0 (5.0, 11.2)	3.62 (1.8, 5.5)
	Rectum	<50	–	–	–	–	–	–	1.53 (-0.5, 3.6)	-0.96 (-5.1, 3.4)
		50+	2001–2004	28.1 (5.3, 55.9)	2004–2016	1.3 (-0.2, 2.8)	–	–	6.20 (2.3, 10.1)	1.87 (-.40, 4.1)
	CRC	<50	–	–	–	–	–	–	1.92 (0.3, 3.6)	-0.15 (-3.4, 3.2)
		50+	2001–2004	28.6 (8.9, 51.9)	2004–2016	2.7 (1.5, 4.0)	–	–	7.5 (4.2, 10.8)	2.90 (1.1, 4.8)
Female	Colon	<50	2001–2007	11.0 (3.3, 19.3)	2007–2016	-1.2 (-4.0, 1.6)	–	–	3.5 (0.4, 6.6)	-1.53 (-4.0, 1.0)
		50+	2001–2006	12.7 (4.3, 21.9)	2006–2016	2.5 (0.7, 4.3)	–	–	5.8 (3.1, 8.5)	2.48 (0.40, 4.6)
	Rectum	<50	–	–	–	–	–	–	0.47 (-1.4, 2.4)	-1.66 (-5.6, 2.4)
		50+	2001–2006	14.6 (5.0, 25.1)	2006–2016	0.2 (-1.8, 2.3)	–	–	4.8 (1.8, 7.8)	0.39 (-1.8, 2.6)
	CRC	<50	2001–2009	5.7 (2.4, 9.2)	2009–2016	-2.4 (-5.4, 0.6)	–	–	1.8 (-0.2, 3.9)	-1.61 (-3.2, 0)
		50+	2001–2006	13.5 (5.9, 21.9)	2006–2016	1.6 (-0.01, 3.3)	–	–	5.5 (3.1, 7.9)	1.70 (-0.3, 3.7)
All	Colon	<50	2001–2007	9.1 (2.8, 15.7)	2007–2016	-0.6 (-2.8, 1.7)	–	–	3.2 (0.7, 5.7)	-0.65 (-2.5, 1.3)
		50+	2001–2006	14.7 (7.4, 22.5)	2006–2016	2.5 (1.0, 4.0)	–	–	6.4 (4.1, 8.7)	2.81 (1.1, 4.6)
	Rectum	<50	–	–	–	–	–	–	0.81 (-1.1, 2.8)	-1.54 (-5.6, 2.7)
		50+	2001–2004	27.5 (7.2, 51.6)	2004–2016	1.0 (-0.3, 2.4)	–	–	5.8 (2.5, 9.3)	0.92 (-1.1, 3.0)
	CRC	<50	2001–2010	5.3 (3.1, 7.5)	2010–2014	-6.7 (-14.7, 2.0)	2014-2016	10.54 (-7, 31.3)	2.6 (-0.4, 5.7)	-1.20 (-3.4, 1.1)
		50+	2001–2005	18.6 (7.5, 30.8)	2005–2016	1.9 (0.6, 3.3)	–	–	6.1 (3.5, 8.8)	2.06 (0.3, 3.9)

While the most pronounced increase in AAPC was among those 50+, early-onset patients have consistent AAPC increase especially among patients diagnosed with colon cancer. The highest increase in AAPC among 50+ was for men diagnosed with colon cancer (AAPC, 8.0 (95% CI 5.0, 11.2)), and the highest increase in AAPC among early-onset was for women diagnosed with colon cancer (AAPC, 3.50 (95% CI 0.4, 6.6)).

**Figure 3:**
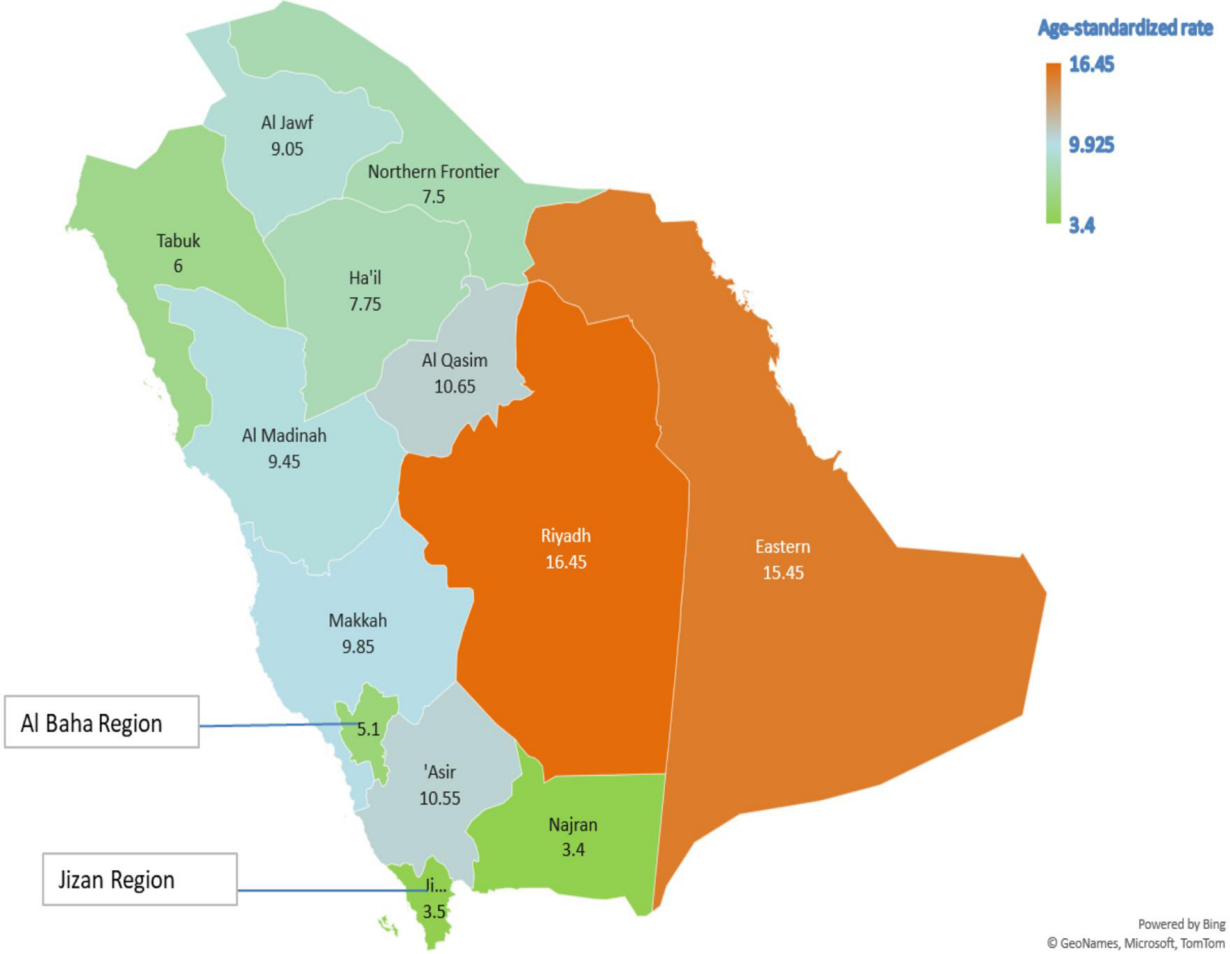
The geographic distribution of the age-standardized incidence rates per 10^5^ population, Saudi Arabia 2016.

**Table 5** presents the AAPC during different periods and across various age groups. Among the early-onset CRC patients, one can notice the persistent increase in the AAPC among the age group 40–49 and males in the age group 30–39. One can also observe the increase in the AAPC among males in almost all age groups. In general, men have a higher increase in AAPC than women (**Table 5** and **Figure 2**), with the highest increase observed in men aged 50–54. Lastly, **Figure 3** shows the geographic distribution of age-standardized incidence rates across Saudi Arabia. The region of Riyadh and the Eastern province had the highest rates, while the regions of Jizan and Najran had the lowest rates in 2016.

**Table 5:** The average annual percentage change in colorectal cancer incidence rates, number of cases, and population at risk of colorectal cancer by gender and age, Saudi Arabia, 2001–2016.

Age group	2001–2016			2007–2016			2012–2016		
	AAPC, (95% CI)	Cases	At risk	AAPC, (95% CI)	Cases	At risk	AAPC (95% CI)	Cases, n	At risk, n
**20–29**									
Male	2.24 (-2.6, 7.3)	213	19496153	-5.97 (-10.62, -1.09)	166	12770676	-10.86 (-12.74, -8.95)	79	6698712
Female	-0.15 (-3.9, 3.7)	203	26917804	-1.51 (-8.98, 6.58)	140	17983467	-0.15 (-3.85, 3.69)	71	9394870
Total	0.61 (-2.4, 3.7)	417	46413957	-3.94 (-7.90, 0.20)	306	30754143	-6.30 (-23.79, 15.20)	150	16093582
**30–39**									
Male	4.16 (2.6, 5.7)	614	18202883	2.45 (0.20, 4.75)	451	11787983	3.11 (-7.92, 15.46)	247	6118127
Female	0.44 (-1.4, 2.3)	655	20040987	0.14 (-2.85, 3.21)	465	13849503	0.44 (-1.43, 2.35)	256	7622327
Total	0.85 (-0.7, 2.4)	1268	38243870	-0.18 (-2.05, 2.47)	916	25637486	6.05 (0.83, 11.53)	503	13740454
**40–49**									
Male	6.0 (4.8, 7.2)	1340	16503235	5.59 (3.19, 8.05)	998	10642711	6.04 (0.43, 11.95)	557	5249650
Female	2.6 (1.1, 4.2)	1391	13315700	-1.95 (-3.80, -0.06)	1057	9388325	2.62 (1.08, 4.18)	565	5328800
Total	3.3 (0.6, 6.0)	2731	29818935	-1.17 (-4.17, 1.93)	2055	20031036	1.11 (-12.15, 16.38)	1122	10578450
**50–54**									
Male	14.40 (6.0, 23.6)	971	14271149	18.85 (6.78, 32.29)	786	9353314	38.84 (0.59, 91.63)	468	4284658
Female	4.04 (1.4, 6.8)	946	4565216	1.48 (-2.48, 5.61)	762	3313250	4.04 (1.38, 6.78)	441	1893713
Total	5.9 (-1.8, 14.2)	1917	18836365	1.96 (-1.65, 5.69)	1548	12666564	5.52 (-8.83, 22.12)	909	6178371
**55–59**									
Male	13.40 (4.4, 23.2)	1117	13134994	20.49 (15.20, 26.02)	899	9041816	33.27 (-7.81, 92.66)	581	4329919
Female	3.79 (1.8, 5.9)	873	3425455	1.47 (-2.12, 5.19)	691	2465862	3.79 (1.76, 5.85)	413	1439765
Total	6.6 (1.4, 12.0)	1990	16560449	2.79 (0.74, 4.87)	1590	11507678	3.63 (-3.02, 10.74)	994	5769684
**60–64**									
Male	12.0 (3.6, 21.2)	1054	11542310	20.67 (13.05, 28.81)	794	7912660	44.62 (-19.98, 161.35)	492	3892314
Female	3.78 (1.9, 5.7)	873	2636916	3.42 (0.29, 6.64)	660	1837727	3.78 (1.88, 5.72)	402	1054044
Total	6.1 (2.0, 10.4)	1927	14179226	2.91 (0.80, 5.06)	1454	9750387	5.40 (-4.62, 16.47)	894	4946358
**65–69**									
Male	5.5 (-0.6, 11.9)	1030	9740352	9.20 (-4.30, 24.61)	773	6821321	41.35 (-25.54, 168.35)	403	3390723
Female	3.27 (-0.1, 6.8)	725	1879501	2.07 (-1.986.29),	557	1327576	3.27 (-0.12, 6.77)	334	753261
Total	5.2 (1.8, 8.8)	1755	11619853	-0.97 (-3.81, 1.96)	1330	8148897	1.13 (-6.23, 9.07)	737	4143984
**70–74**									
Male	14.2 (6.5, 22.5)	933	8233623	23.88 (18.66, 29.34)	713	5744946	56.25 (-30.29, 250.22)	436	2921751
Female	6.0 (2.5, 9.6)	601	1399651	0.07 (-3.51, 3.78)	468	926243	6.01 (2.52, 9.63)	267	514943
Total	8.5 (5.1, 12.1)	1534	9633274	3.83 (1.71, 6.0)	1181	6671189	-0.02 (-6.99, 7.46)	703	3436694
**75+**									
Male	12.0 (5.1, 19.5)	1374	7002390	17.22 (9.06, 25.99)	1081	4860583	48.44 (-21.01, 178.93)	633	2515466
Female	3.27 (0.7, 5.9)	914	1884621	0.74 (-4.03, 5.75)	705	1323235	3.27 (0.75, 5.86)	397	726320
Total	7.0 (2.6, 11.6)	2288	8887011	2.45 (-1.14, 6.18)	1786	6183818	4.53 (-10.71, 22.36)	1030	3241786

Lastly, the supplementary materials show the comparison in incidence rate trends between colon and cancer patients by age at diagnosis (<50 *vs*. 50+ years old). Overall, there has been a significant increase in the rates of colon cancer especially in 50+ years old patients, with a slower increase in patients with rectal cancer diagnosis (**Supplementary Figures**).

## Discussion

The present study was designed to elucidate the magnitude and direction of CRC incidence rate trends by age and gender in the Saudi population. We found that both the early- and late-onset CRC incidence rates have been increasing during the past years, with the steepest increase in the age group 40–49. While there is a lack of local and regional incidence rate trends data, one study estimated the APC during 1999 and 2003 in the Saudi population (21). Authors found significant increases in APC among males (20.5% during 1999–2003) and a non-significant increase among females (6.06% during 2001–2003). Our regression models showed comparable results in males (18.01% during 2001–2005) and females (12.30% during 2001–2006). Taken together, local data suggest increases in all age groups with a more pronounced increase in males.

Similarly, international studies have shown increases in CRC incidence rates among the younger age group. Although CRC is frequently diagnosed in the elderly population (22), data from different countries have shown an alarming increase in the incidence of early-onset CRC amongst men and women (23–25). Between 2008 and 2012, the AAPC in the incidence of early-onset CRC was documented at 4, 2.8, 2.8, and 2.2% in New Zealand, Canada, Australia, and the USA, respectively (3). As indicated above, there is limited data reported on this subject in the GCC countries. For instance, a limited analysis of 387 CRC cases in the United Arab Emirates (UAE) diagnosed in 2015 reported that 41.9% were diagnosed at an age younger than 50 years (26). Only less than one-third of the population from this analysis were UAE nationals. Given the small number of patients, and the heterogeneity of the reported population, it may be difficult to draw conclusions about the trends of early-onset CRC.

Unlike other countries where the incidence of late-onset CRC has declined or remained stable (27), we have shown that the incidence of late-onset CRC in Saudi Arabia continued to rise. Moreover, less than one-third of Saudi patients were diagnosed with localized disease (14). This is likely related to the lack of an effective national screening program for CRC. Our analysis showed that there is an incremental increase of both early- and late-onset CRC. The AAPC of early-onset CRC in our report is comparable to western populations.

As we have shown, increases in incidence rates amongst young individuals (<50 years) have not been the same between the colon and rectal cancer cases in Saudi Arabia. The degree of increase has been less for rectal cancer when compared with colon cancer cases (**Supplementary Figures**). This is, in part, maybe due to differences in risk factors with certain ones affecting colon rather than rectal cancer. Dietary changes, lack of physical activity, and alcohol intake are known and established risk factors for colon cancer, but not for rectal cancer (28). Obesity was also shown in a meta-analysis to be a risk factor for colon cancer; however, this association was not seen in women with rectal cancer (29). Obesity during childhood has been associated with the increase in early-onset CRC (30). Furthermore, the observation of differences between colon and rectal cancer rates was also reported by a large study on a European population by Vuik and colleagues (19).

Trends in CRC incidence rates in Saudi Arabia reported in this study are in line with what has been reported by other studies in USA (31), Europe (19), and the UAE (26). These results have major implications on our population, healthcare system, and other involved stakeholders. This increase in early-onset CRC resulted in major oncology societies like the American Cancer Society, in 2018, lowering the age for screening for CRC to the age of 45 years (32). The results of their modeling assumed that screening those between the ages of 45–50 would have a preventive effect just as screening those above 50 years of age. Their analyses showed that this will result in a reduction in incidence and mortality, and that benefit-burden balance is favoring screening this younger group. More recently, the USPSTF recommended that adults from the age of 45 years get screened for CRC. Targeting this younger population is therefore important in our population.

Moving forward with establishing a national screening program with a special focus on young-onset CRC is essential. A large survey of more than 1000 young-onset CRC patients reported that more than half were diagnosed at a later stage (stages III & IV), needed more time to be diagnosed, and visited more than two physicians before a correct diagnosis of CRC was made (33). Therefore, education of health care providers on being vigilant and aware of signs and symptoms of CRC regardless of age is crucial. This needs engagement from health agencies, medical societies, and perhaps media in a collaborative national effort to address the needs of this vulnerable group of young-onset CRC patients. Studying and, more importantly, addressing modifiable risk factors of early-onset CRC such as obesity, diet, and lack of exercise are needed. Furthermore, a comprehensive survivorship cancer care and availability of genetic testing are needed to improve the care of young patients with CRC.

One of the strengths of the present study is the use of SCR, which is based on all regions of Saudi Arabia and is therefore representative and generalizable to the Saudi population. Moreover, the current study leveraged the lengthy period of data collection which includes all CRC cases in Saudi Arabia and thus utilized the latest available 16 years of CRC data, the longest studied period in CRC Saudi patients. In addition to the strengths, several limitations should be considered when interpreting the results of our study. First, the current study lacks tumor-sidedness data which has prognostic value, and also lacks molecular characterization of tumors. These variables are not available in the original data source and therefore were not investigated. Second, we were unable to assess patient-level information, which includes variables such as education, income, and other sociodemographic variables because our analysis was based on aggregate data. Nonetheless, aggregate data are very useful for assessing trends in cancer rates (34). Third, patients, especially residents of remote/rural areas, are referred to a tertiary hospital in major cities for cancer care and could be a potential source of referral bias, which might result in underestimation of cases in remote areas. Nevertheless, this kind of selection bias is less likely to affect the findings of the present study given the case ascertainment method implemented by the SCR (35). Fourth, we lack contemporary data (2017–2019) in this study because the year 2016 is the latest available year reported by the SCR. Lastly, our data show an increase in CRC during the year 2016 compared to earlier years. The sudden increase in incidence rate could be due to the implementation of opportunistic CRC (*vs*. organized or population-based) screening, after the initial publication of CRC guidelines in 2015 (36).

## Conclusion

Both early-onset and late-onset CRC are increasing in Saudi Arabia. For early-onset CRC, primary health care providers must be alerted about the increasing rate and should possibly investigate the cancer family history in the younger population, especially in Saudis aged 30–49 years who had the highest increase in CRC incidence. Additionally, national efforts directed to prevention measures such as CRC screening are warranted.

## Data Availability Statement

Publicly available datasets were analyzed in this study. This data can be found here: https://nhic.gov.sa/eServices/Pages/TumorRegistration.aspx.

## Author Contributions

MeA designed the study, analyzed the data, discussed the results, and wrote the manuscript. MoA and KA discussed the results, searched the literature, wrote the manuscript, and collected data. All authors approved the final version of the manuscript. All authors agree to be accountable for all aspects of the work.

## Conflict of Interest

The authors declare that the research was conducted in the absence of any commercial or financial relationships that could be construed as a potential conflict of interest.

## Publisher’s Note

All claims expressed in this article are solely those of the authors and do not necessarily represent those of their affiliated organizations, or those of the publisher, the editors and the reviewers. Any product that may be evaluated in this article, or claim that may be made by its manufacturer, is not guaranteed or endorsed by the publisher.

## Abbreviations

APC, Annual Percent Change; AAPC, Average Annual Percent Change; CRC, Colorectal Cancer; GCC, Gulf Cooperation Council; IARC, International Agency for Research on Cancer; ICD, International Classification of Diseases; SCR, Saudi Cancer Registry; SEER, Surveillance, Epidemiology, and End Results; UAE, United Arab Emirates; USPSTF, United State Preventive Services Task Force.
