# Supplementary material for: Trends in Colorectal Cancer Incidence Rates in Saudi Arabia (2001–2016) Using Saudi National Registry: Early- Versus Late-Onset Disease
Source: Front Oncol. 2021 Sep 9;11:730689. doi: 10.3389/fonc.2021.730689 (PMC8460085; doi:10.3389/fonc.2021.730689)

Trends in Colorectal Cancer Incidence Rates in Saudi Arabia (2001-2016) Using Saudi National Registry: Early- versus late-onset disease

Mesnad Alyabsi, MS, MBA, Ph.D.

Mohammed Algarni, MD

Kanan Alshammari. MD

Supplementary Figures. Trends in colon cancer patients <50 years old (A), rectal cancer patients <50 years old (B), colon cancer patients 50+ years old (C) and rectal cancer patients 50+ years old (D).


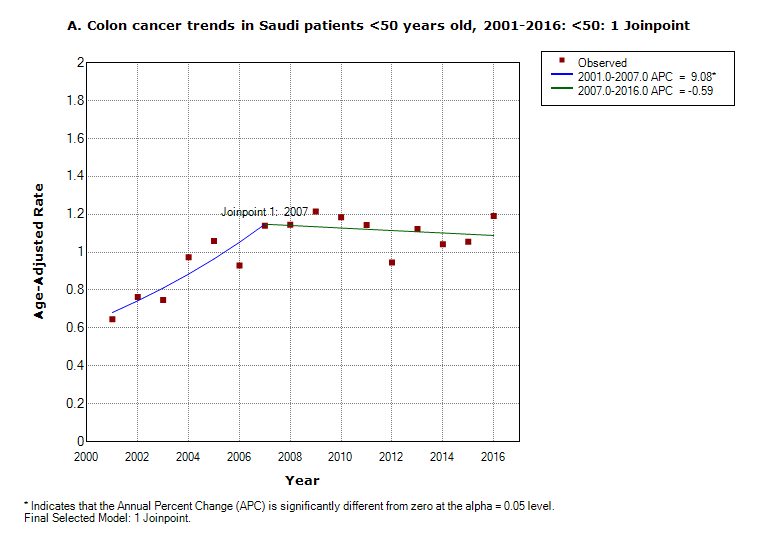


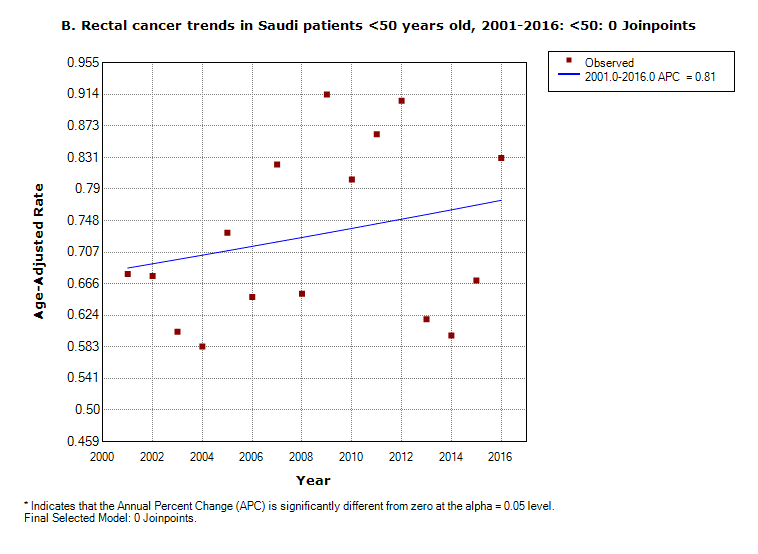


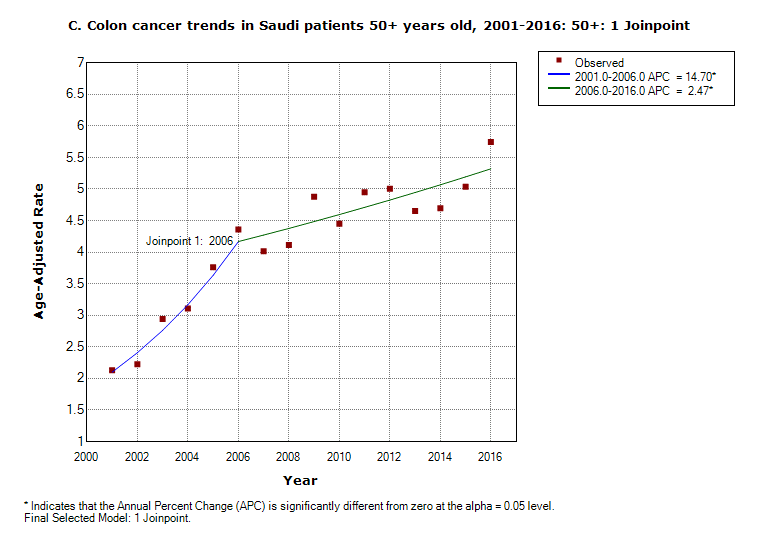


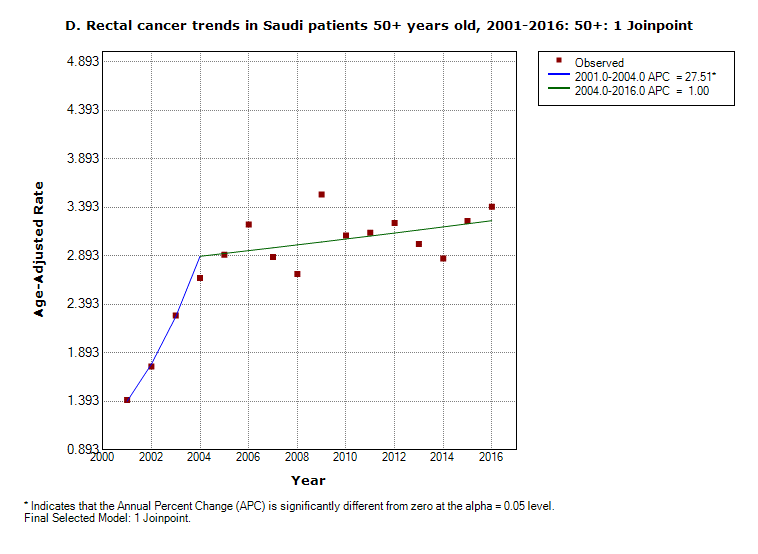

Supplement: Supplementary file 1 [file DataSheet_1.docx]
